# Genome-wide association analysis of bean fly resistance and agro-morphological traits in common bean

**DOI:** 10.1371/journal.pone.0250729

**Published:** 2021-04-29

**Authors:** Wilson Nkhata, Hussein Shimelis, Rob Melis, Rowland Chirwa, Tenyson Mzengeza, Isack Mathew, Admire Shayanowako

**Affiliations:** 1 African Centre for Crop Improvement, School of Agricultural, Earth and Environmental Sciences, University of KwaZulu-Natal, Scottsville, Pietermaritzburg, South Africa; 2 Alliance of Biodiversity International and CIAT, Chitedze Agricultural Station, Lilongwe, Malawi; 3 Department of Agricultural Research Service, Chitedze Agricultural Research Station, Lilongwe, Malawi; University of Delhi, INDIA

## Abstract

The bean fly (*Ophiomyia* spp) is a key insect pest causing significant crop damage and yield loss in common bean (*Phaseolus vulgaris* L., 2n = 2x = 22). Development and deployment of agronomic superior and bean fly resistant common bean varieties aredependent on genetic variation and the identification of genes and genomic regions controlling economic traits. This study’s objective was to determine the population structure of a diverse panel of common bean genotypes and deduce associations between bean fly resistance and agronomic traits based on single nucleotide polymorphism (SNP) markers. Ninety-nine common bean genotypes were phenotyped in two seasons at two locations and genotyped with 16 565 SNP markers. The genotypes exhibited significant variation for bean fly damage severity (BDS), plant mortality rate (PMR), and pupa count (PC). Likewise, the genotypes showed significant variation for agro-morphological traits such as days to flowering (DTF), days to maturity (DTM), number of pods per plant (NPP), number of seeds per pod (NSP), and grain yield (GYD). The genotypes were delineated into two populations, which were based on the Andean and Mesoamerican gene pools. The genotypes exhibited a minimum membership coefficient of 0.60 to their respective populations. Eighty-three significant (P<0.01) markers were identified with an average linkage disequilibrium of 0.20 at 12Mb across the 11 chromosomes. Three markers were identified, each having pleiotropic effects on two traits: *M100049197* (BDS and NPP), *M3379537* (DTF and PC), and *M13122571* (NPP and GYD). The identified markers are useful for marker-assisted selection in the breeding program to develop common bean genotypes with resistance to bean fly damage.

## Introduction

Common bean (*Phaseolus vulgaris* L., 2n = 2x = 22) is one of the most important grain legume crops globally. The crop is cultivated for food, cash income, and improves soil fertility through biological nitrogen fixation [1,2]. It is a primary source of dietary protein for many people in Latin America and sub-Saharan Africa (SSA) [3,4]. Common bean provides 15% of daily calorie intake globally, while it provides 36% of protein intake in Africa [5]. The common bean is a source of micronutrients such as folic acids, iron, thiamine, and zinc [6,7]. Approximately 40% of the bean produced in eastern and southern Africa (ESA) is marketed in local or regional outlets, providing a total monetary value of about US$ 450 million per annum [8]. Common bean fixes atmospheric nitrogen through their symbiosis with the nitrogen-fixing bacteria *Rhyzobium*. The common bean’s ability to fix nitrogen is valued in the legume-cereal crop rotation systems (e.g. maize-common bean system) among smallholder farmers in SSA to improve soil fertility [9].

The global production of common bean is approximately 30 million tonnes per annum, with the bulk of the production occurring in Latin America and ESA [10]. The total production of common bean in ESA falls below demand due to low productivity (<600 kg/ha) caused by several constraints, including insect pests, diseases, drought and heat, and a lack of improved cultivars [11]. One of the major insect pests of common bean in ESA is the bean fly (*Ophiomyia* spp), belonging to the *Agromyzidae* family [12]. The bean fly causes up to 100% yield losses [13].

Both the adult and larvae of the bean fly cause significant crop damage. However, the larvae cause the most significant damage [14,15]. After oviposition under the surface of young bean leaves, larvae burrow under the thin layer of the leaf (epidermis) and tunnel along the veins down to the stem and lodges where the stem touches the soil. Pupation takes place inside the bean stem, resulting in swelling and cracking of the stem at the point where the pupae are lodged, which destroys the transport system of nutrients from the roots and products of photosynthesis from the leaves, leading to stunted growth, and yellowing of leaves at an early plant stage. Heavily infested crop stands are characterised by premature leaf drop and plant death [13,15]. The bean fly is widely distributed in all bean growing agro-ecologies in Malawi and is considered the main cause of low yields. Thus far, the country’s common bean varieties are highly susceptible to the bean fly pest [16].

The most widely recommended control methods of the bean fly pest include early planting, intercropping, spraying with organophosphate insecticides, and cultivating resistant cultivars [17,18]. The use of host plant resistance is regarded as the most sustainable method to manage insect pests and diseases. Over the past three decades, few bean fly resistance sources in common bean have been identified [19–21]. However, the utilisation of the genetic resources has been limited, and there are only a few commercial cultivars with bean fly resistance [22]. The scarcity of information on the genetic basis of bean fly resistance has contributed to the slow progress in breeding and development of cultivars with improved bean fly resistance [22]. Bean fly resistance is a complex trait and conditioned by polygenes with major and minor genetic effects [23,24]. It is essential to identify genetic markers associated with bean fly resistance and important agronomic traits such as reduced maturity period, pod and grain yields in common bean, and integrate marker-assisted and conventional breeding techniques for accelerated breeding and genetic gain [25].

Genome-wide association studies (GWAS) based on linkage disequilibrium (LD) complements conventional linkage mapping to identify genomic regions controlling quantitative traits of interest [26]. GWAS has been used in common bean to identify genes associated with cooking time [27], days to flowering [28], yield-related traits [29], and resistance to angular leaf spot [30], anthracnose [31], common bacterial blight [32], soybean cyst nematodes [33,34], bruchids [35] and bean fly [36]. [Ojwang, Eldridge [36]] identified five significant SNP markers linked to bean fly resistance in common bean on chromosome *Pv01*, and one of the markers was reportedly linked to an immune response gene *PHAVU_001G075500g* [37]. This was the first identified gene associated with bean fly resistance [36].

To design and deploy superior, farmer-preferred, and bean fly resistant common bean varieties adapted to Malawi conditions, diverse common bean genetic resources were collected from different sources. This study aimed to determine the population structure of a diverse panel of common bean genotypes and deduce associations between bean fly resistance and agronomic traits based on single nucleotide polymorphism (SNP) markers.

## Materials and methods

### Germplasm

A total of 99 common bean genotypes were used in the study. Of these genotypes, forty-two were landraces sourced from the genetic resource unit, under the Department of Agricultural Research Services (DARS) in Malawi, and the Tanzania Agricultural Research Institute–Uyole (TARI-Uyole). Forty-one (41) genotypes were breeding lines sourced from the Agricultural Research Council of South Africa (ARC), the Kenya Agriculture and Livestock Research Organisation (KARLO), and the International Centre for Tropical Agriculture (CIAT). Sixteen (16) genotypes were released varieties sourced from the Malawi National Bean Improvement Program and CIAT bean improvement program in Malawi. The panel consisted of bean fly resistant lines A429, A55, and Sinoni [38], which were used as checks.

### Study sites and experimental design

The genotypes were evaluated for bean fly resistance and agronomic traits at Chitedze (13.85° S; 33.38° E) and Mbawa (12.06° S; 33.25° E) Agricultural Research Station farms in Malawi during the 2018 and 2019 growing seasons. Both locations receive unimodal rainfall, and the main rainy season falls between November and April. The mean annual rainfall for Chitedze and Mbawa sites is 900 mm and 692 mm, respectively. Mean monthly temperature ranges from 16°C in July to 24°C in November at Chitedze, and from 16.5°C in July to 26°C in October at Mbawa. The soil at the Chitedze experimental farm is deep brown loam soil, while Mbawa is characterised by red clay soils [39]. The genotypes were established using a 9 × 11 alpha lattice design with three replications. Each genotype was planted in 3 m two-row plot spaced at 75 cm. Bean seeds were planted at 10 cm apart. Standard agronomic practices were carried during plant growth [16]. Chitedze and Mbawa are hot spots for bean fly, hence they were selected for the study [16].

### Phenotypic data collection and analysis

Bean fly severity damage (BSD) was evaluated based on overall plot damage scores using a severity rating scale (1 to 9) where scores between 1 and 3 represented high resistance, 4 to 6 moderate resistance, and 7 to 9 defined susceptibility, as described by Corrales and van Schoonhoven [40]. The PC was scored two weeks after germination through destructive sampling using four randomly selected plants in each plot. The plant mortality rate (PMR) was calculated as a percentage of the number of dead plants in a plot due to bean fly attack [24,41]. Data were also recorded on five agronomic traits. Days to 50% flowering (DTF), days to 90% maturity (DTM), number of pods per plant (NPP) from five tagged plants per plot at harvest, number of seeds per pod (NSP) from five tagged plants in a plot at harvest. Grain yield (GYD) was recorded as the weight of dried seeds converted to kilograms per hectare. The description of data collection is fully explained in [42].

The data on bean fly resistance and agronomic traits were subjected to an analysis of variance using GenStat 18^th^ edition. The means were separated by the Fisher’s protected least significant difference at 5% probability [43].

### Genotyping

The 99 genotypes were planted in seedlings trays and raised to the three-leaf growth stage in a greenhouse. Fresh and young leaves were harvested for genomic DNA extraction. Genomic DNA was extracted following the plant DNA extraction protocol adapted from [41]. After extraction, DNA quality was checked for nucleic acid concentration and purity using a NanoDrop 2000 spectrophotometer (ND-2000 V3.5, NanoDrop Technologies Inc). The genomic DNA was shipped to the Biosciences Eastern and Central Africa (BecA) Hub of the International Livestock Research Institute (BecA-ILRI) in Kenya for genotyping by sequencing. The Diversity Array Technology Sequencing (DArTseq) protocol was used for genotyping the samples using 17,190 SNP markers assigned to 11 chromosomes of the common bean. The SNP markers used had a reproducibility value of 1, polymorphic information content (PIC) values ranging from 0.020 to 0.50, and a mean call rate of 0.93 ranging from 0.84 to 1.00. A total of 15 565 SNP markers and 93 genotypes were used after data imputation where SNP loci and individuals with <20% missing data and rare SNP, with <5% minor allele frequency (MAF) were pruned from the data before analysis following Mathew et al., [44].

### Genetic diversity and population structure analysis

The genomic data were imputed using the optimal imputation algorithm on the KDCompute sever (*https*:*//kdcompute*.*igss-africa*.*org/kdcompute/*). The SNP distribution across the genome was visualized graphically in KDCompute. The polymorphic information content (PIC), minor allele frequency (MAF), observed heterozygosity (H_o_), genetic distance (GD), were estimated using the R package “adegenet” [45]. The genomic data were subjected to population structure analysis in STRUCTURE version 2.3.4 based on the Bayesian clustering method [46]. The burn-in period and Markov Chain Monte Carlo (MCMC) iterations were set at 20,000. The number of clusters (K) was estimated to be between 1 and 10, and the best K-value was determined by the Evanno method based on ΔK in CLUMPAK [47]. The 3D visualization of the genotype clustering was conducted using the principal component analysis (PCA) in prcomp R 3.0 function [48,49].

### Marker-trait association and linkage disequilibrium analyses

Before association mapping, best linear unbiased predictors (BLUPs) were derived for the phenotypic traits using the random mixed model in DeltaGen software [50]. BLUPs minimise the effects of the environmental and seasonal variations, which eliminates the need to conduct marker-trait association for each environment independently [44]. The BLUPs were used as input in the GWAS analysis. Association mapping was performed using the association compress mixed linear model (CMLM) following the Q + K model that utilizes both population structure (Q) and kinship matrix (K) in the GAPIT program of R software following [44,51]. The p values and false discovery rate thresholds were set at 0.001 and 0.05, respectively. The log10 (p) value distributions were displayed in quantile-quantile (q-q) plots and Manhattan plots using the mhtplot function R package gap [52]. The linkage disequilibrium (LD) was deduced in the GAPIT program of R software [51]. The LD Heatmaps were generated in R 3.0 using the LDHeatmap package [53] in R package [48] for each trait’s significant markers. The significant markers for each trait were blasted on Ensemble based on the *Phaseolus* reference genome to identify putative genes associated with the markers. Gene ontology of the potential candidate genes was conducted using SMARTBLAST https://blast.ncbi.nlm.nih.gov/smartblast/smartBlast.cgi.

## Results

### Phenotypic diversity analysis

The combined analysis of variance revealed that genotype × location × year interaction effects were significant (p<0.05) for BDS, PC, DTF NPP, and GYD (Table 1). In addition, BDS, PMR, DTF, DTM, NPP, NSP and NSP were significantly (p < 0.05) impacted by the genotype × location interaction effects. The genotype × year interaction effects were significant (p < 0.05) for BDS, PC, PMR, DTF, NPP, NSP and GYD. All the agronomic traits and bean fly parameters except PM exhibited variability due to the main effect of genotype, location, and year. Overall, BDS ranged from 1 to 8 with a mean of 6.56 (Table 2). Plant mortality due to bean fly ranged from 10 to 87 percent. Breeding lines exhibited higher resistance to bean fly compared to landraces and released varieties. Overall, Mesoamerican genotypes exhibited a higher level of resistance than the Andean genotypes. For instance, the mean BDS score for Mesoamerican genotypes was 4.89, while it was 6.08 for Andean genotypes. The PMR ranged from 10 to 63 percent among the Mesoamerican genotypes, while it ranged from 19 to 87 percent for the Andean genotypes. Overall, DTF ranged from 35 to 45 with a mean of 41, while DTM ranged from 69 to 90 with a mean of 78 and GYD ranged from 158 to 1133 kg/ha with a mean of 371.73 kg/ha. When the genotypes were separated by groups, the mean grain yield for breeding lines was higher (438.10 kg/ha) compared to landraces (329.98 kg/ha) and released varieties (311.25 kg/ha).

**Table 1:** Mean squares and significant tests for three bean fly resistance parameters and five agro-morphological traits among 99 common bean genotypes assessed in two locations and two years in Malawi.

Source of variation	DF	BDS	PC	PMR	DTF	DTM	NPP	NSP	GYD
Replication	2	4.40	35.5***	2392.69*	38.75	60.55	51.24**	0.54	479419.95***
Replication (block)	24	3.28	5.06	841.76	16.79	136.60***	12.79	0.79	36204.26
Genotype (G)	98	22.91***	6.18*	2371.41***	53.24***	188.31***	69.60***	1.65***	351422.28***
Location (L)	1	47.75**	710.41***	31005.91***	6018.40***	1815.46***	975.02***	11.19***	4956546.91***
Year (Y)	1	11.69	462.89***	25394.72***	2322.85***	10782.54***	22.79	15.91	371603.72**
G x L	98	13.51***	4.87	1445.16***	40.69***	122.27****	27.79***	1.00***	96487.32***
G x Y	98	5.23*	6.87**	837.26***	42.62***	52.85	25.56***	0.64***	89709.96***
Y x L	1	62.48***	199.65***	1252.43	3412.16***	10660.82***	1914.64**	1.25***	400766.8**
G x L x Y	98	6.8***	7.45***	754.97	21.57	45.47	28.76***	0.79**	82167.65***
Error	758	3.72	4.92	681.77	14.93	58.92	13.31	0.70	45489.90

DF = degrees of freedom, G × Y = genotype x year interaction, G × L = genotype by location interaction, L × Y = location by year interaction, G × L × Y = genotype x location x year interaction, BDS = bean fly damage severity, PC = pupa count, PMR = plant mortality rate, DTF = days to 50% flowering, DTM = days to 90% physiological maturity, NPP = number of pods per plant, NSP = number of seed per pod and GYD = grain yield.

*, ** and *** denote significant differences at P < 0.05, P<0.01 and P<0.001, respectively.

**Table 2:** Summary statistics for three bean fly resistance parameters and five agronomic traits when evaluating 99 common bean genotypes in two locations (Chitedze and Mbawa research stations) and two years (2018 and 2019) in Malawi.

Population	Parameter	BDS	PC	PMR (%)	DTF	DTM	NPP	NSP	GYD
Breeding line (N = 41)	Minimum	1.00	2.00	10.00	36	73	6	4	162.00
	Maximum	8.00	5.00	73.00	45	85	19	6	1133.00
	Mean	5.07	3.32	34.39	42	78	11	5	438.10
Landrace (N = 42)	Minimum	2.00	2.00	19.00	35	69	6	4	158.00
	Maximum	8.00	5.00	81.00	45	87	19	6	1081.00
	Mean	6.02	3.86	43.57	41	78	9	5	329.98
Released varieties (N = 16)	Minimum	4.00	2.00	30.00	37	69	7	4	172.00
	Maximum	8.00	5.00	87.00	44	90	15	6	547.00
	Mean	6.13	3.25	47.69	41	79	9	5	311.25
Andean (N = 63)	Minimum	2.00	2.00	19.00	36	69	6	4	158.00
	Maximum	8.00	5.00	87.00	45	90	19	6	1081.00
	Mean	6.08	3.63	44.63	41	78	9	5	316.43
Mesoamerican (N = 36)	Minimum	1.00	2.00	10.00	35	70	6	4	162.00
	Maximum	8.00	5.00	73.00	45	89	19	6	1133.00
	Mean	4.89	3.36	33.08	42	79	12	5	468.50
Total (N = 99)	Minimum	1.00	2.00	10.00	35	69	6	4	158.00
	Maximum	8.00	5.00	87.00	45	90	19	6	1133.00
	Mean	5.65	3.54	40.43	41	78	10	5	371.73
	CV%	34.20	62.75	63.61	9.24	9.70	35.10	16.98	54.97

N = number of genotypes, BDS = bean fly damage severity, PC = pupa count, PMR = plant mortality rate, DTF = days to 50% flowering, DTM = days to 90% physiological maturity, NPP = number of pods per plant, NSP = number of seed per pod, GYD = grain yield and CV = Curriculum vitae.

### Population structure and diversity analysis

**Fig 1:**
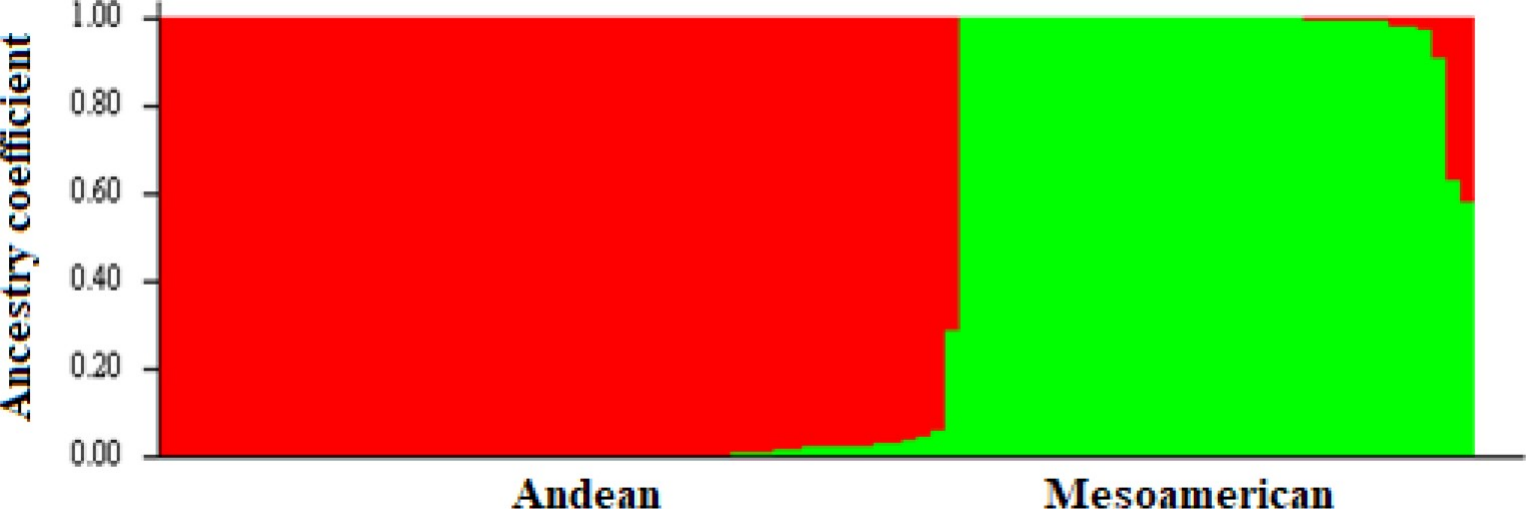
Population structure of 99 common bean genotypes based on SNP markers (K = 2). Red indicates Andean subpopulation and green represent Mesoamerican subpopulation.

**Fig 2:**
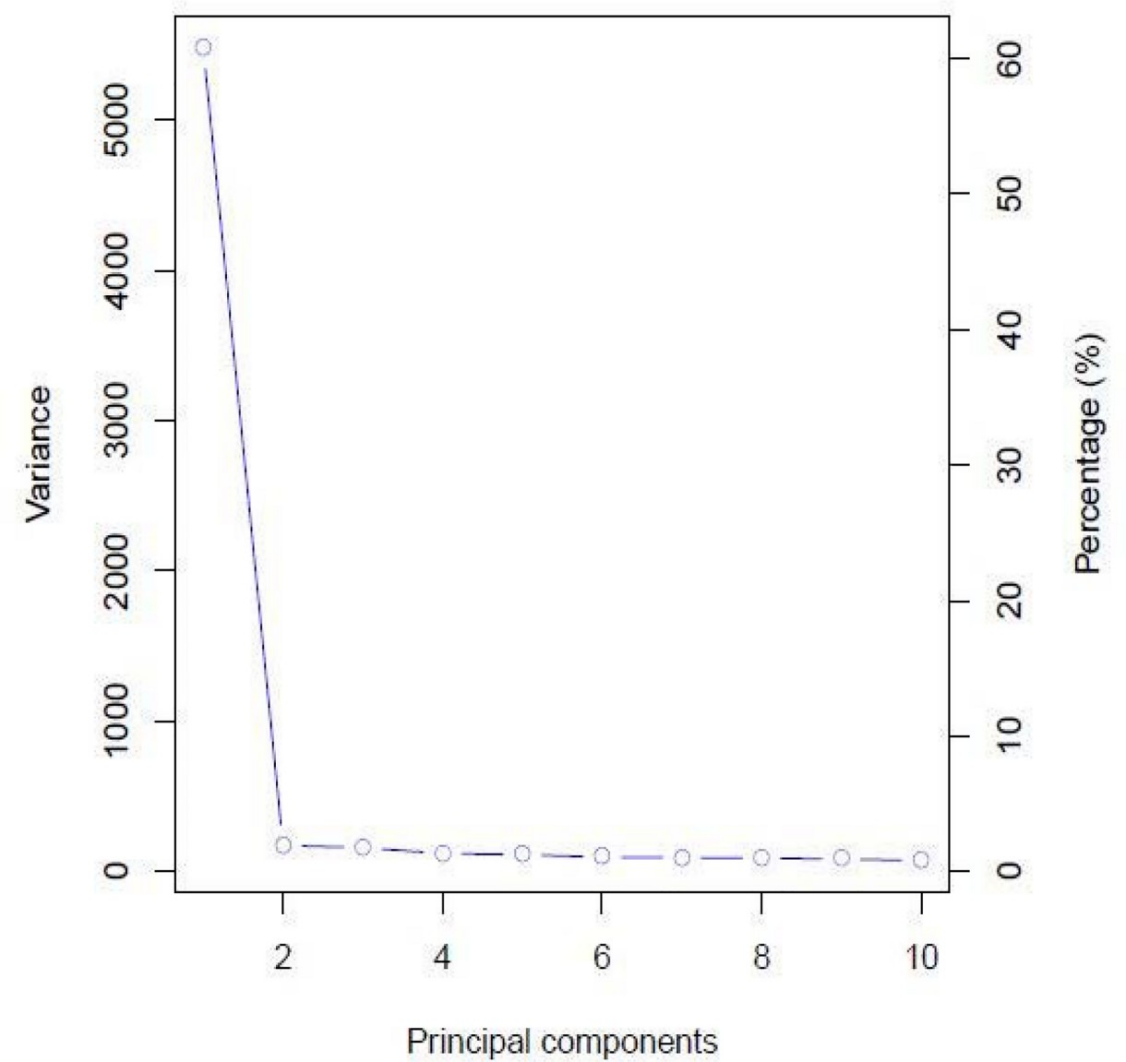
Scree plot showing principal components when assessing 99 common bean genotypes using SNP markers.

**Fig 3:**
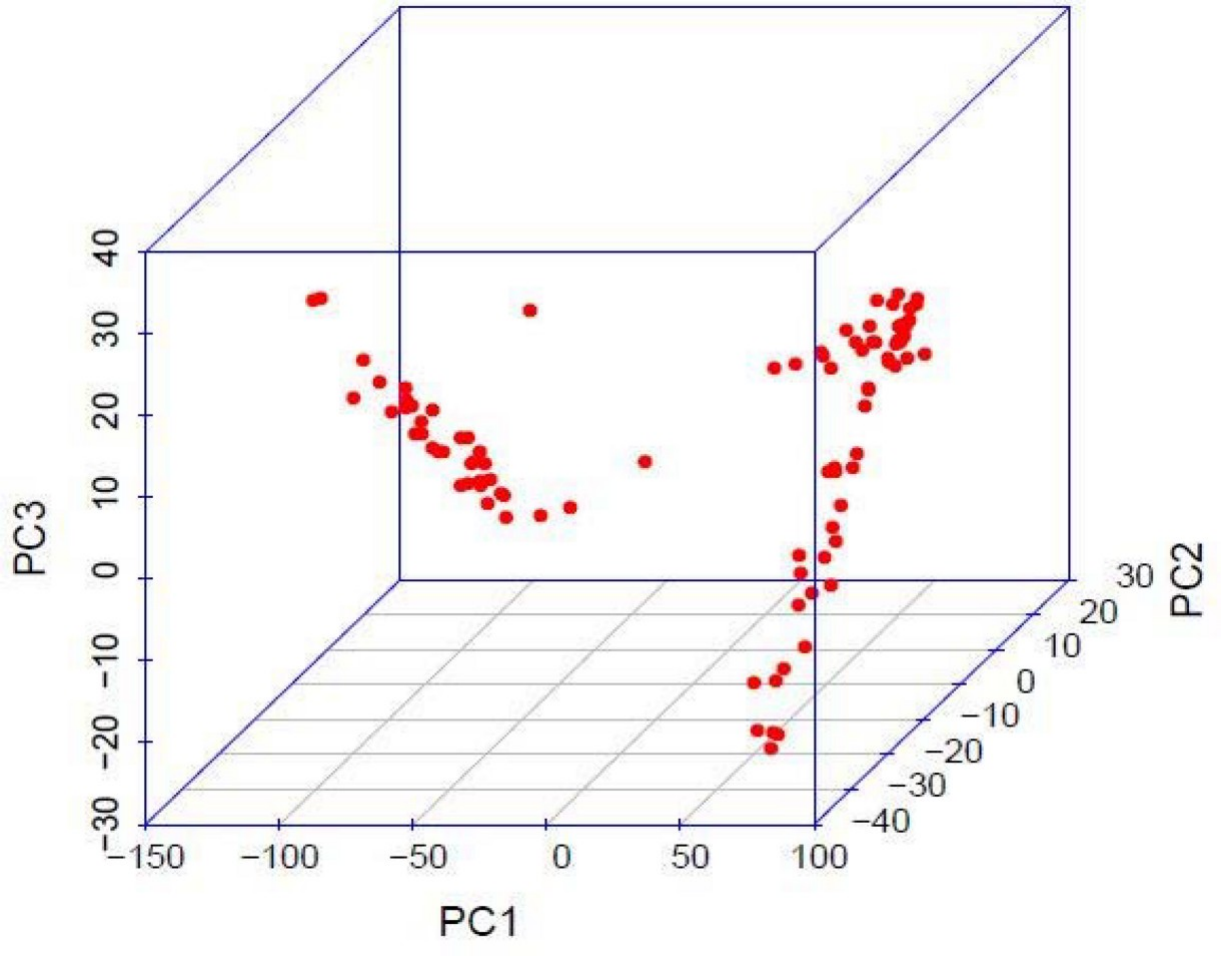
Three dimensional principal coordinate analysis when assessing 99 common bean genotypes with SNP markers.

The highest value for ΔK occurred at K = 2, showing that the genotypes could be delineated into two sub-populations (Fig 1). The minimum coefficient for membership to a particular sub-population was 0.60. The two sub-populations were based on the Andean and Mesoamerican gene pools, and approximately over 60% of the genotypes were from the Andean gene pool. The first principal component accounted for more than 60% of the genotypes’ variation, while the second and third principal components accounted for less than 5% each (Fig 2). The principal component analysis clustered the genotypes into two distinct groups, congruent with the structure analysis (Fig 3). The SNP markers used included rare variants with a minimum MAF of 0.05 and common variants with a maximum MAF of 0.5 with a mean MAF of 0.23 (Table 3). The mean polymorphic information content (PIC) of the markers was 0.25, varying between 0.01 and 0.38. The heterozygosity ranged between 0.21 and 0.45 with a mean of 0.38.

**Table 3:** Estimates of genetic diversty parameters of 99 common bean genotypes gneotyped with 16,565 SNP markers.

Parameter	MAF	GD	H_o_	PIC	F
Minimum	0.01	0.01	0.21	0.01	-0.43
Maximum	0.50	0.50	0.45	0.38	0.34
Mean	0.23	0.31	0.38	0.25	-0.23

MAF = minor allele frequency, GD = the Genetic distance, Ho = the Observed heterozygosity, PIC = the polymorphic information content.

### Significant marker-trait associations

**Fig 4:**
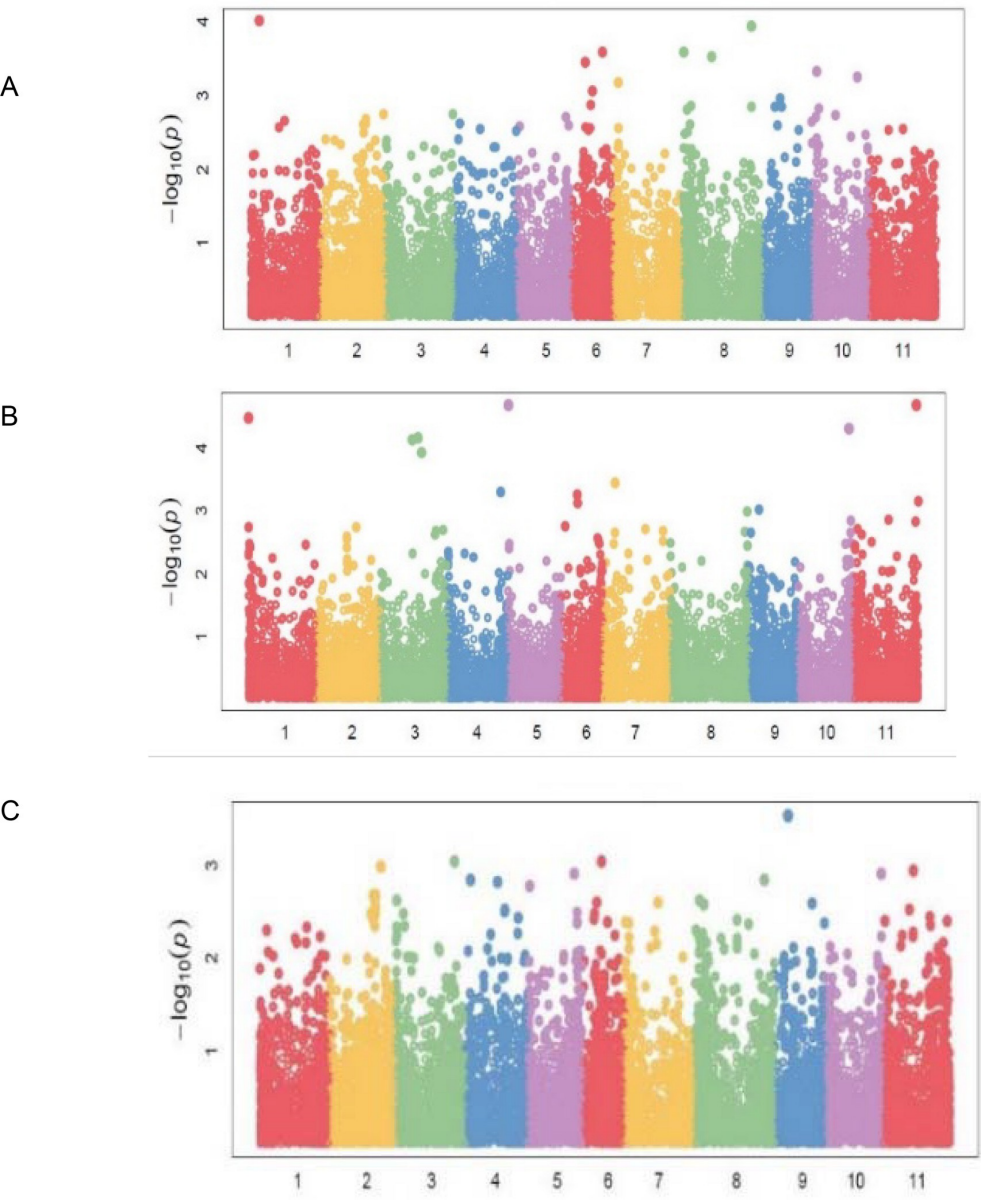
Manhattan plots showing candidate single nucleotide polymorphism and their probability values from genome wide association of 99 common bean genotypes assessed in two locations (Chitedze and Mbawa research stations) and two years (2018 and 2019) in Malawi. Note: A = Bean fly damage severity (BDS), B = Pupa count (PC), C = Plant mortality rate (PMR).

Eighty-three (83) significant (P<0.001) marker-trait associations (MTAs) were identified for all the traits (Table 4). The quantile-quantile (QQ) plots (S1A–S1C, S2A, S2B and S3A–S3C Figs) for all the traits showed that the expected and observed probability values conformed to normal distribution. Significant markers associated with bean fly resistance traits were evenly distributed across the 11 chromosomes. For BDS, the markers were identified on chromosomes *Pv01*, *Pv06*, *Pv07*, *Pv08*, and *Pv10* (Table 4; Fig 4A). Chromosomes *Pv06* and *Pv08* had three markers each that had a significant association with BDS. Markers *M3381188* on *Pv01* and *M3383205* on *Pv08* were strongly associated with BDS exhibiting R^2^ values of about 0.36 (Table 4). Markers associated with PC were identified on all chromosomes except *Pv02* and *Pv08* (Table 4; Fig 4B). Chromosome *Pv03* had three markers associated with PC, followed by *Pv06* and *Pv11*, which had two markers each. Markers associated with PMR were identified on *Pv03*, *Pv06*, and *Pv09* (Table 4; Fig 4C).

**Table 4:** Significant markers for bean fly resistance and agronmic traits and putative genes identified in 93 common bean genotypes assessed in two locations (Chitedze and Mbawa research stations) and two years (2018 and 2019) in Malawi.

Trait	SNP	Chromosome	Position	P-Value	MAF	R square	Candidate gene
BDS	3381188	1	6465142	0.000	0.39	0.37	
	3383205	8	52033029	0.000	0.31	0.37	
	3375408	8	1235760	0.000	0.40	0.35	
	100077834	6	24103936	0.000	0.35	0.35	
	100049197	8	22139614	0.000	0.22	0.34	
	3383436	6	11110706	0.000	0.37	0.34	
	13122350	10	3982570	0.000	0.27	0.33	
	100116632	10	34395982	0.00	0.36	0.33	
	3372405	7	3947278	0.00	0.23	0.33	
	8198832	6	16395421	0.00	0.37	0.32	
PC	3377482	5	413821	0.000	0.02	0.23	PHAVU_005G005200g
	8209338	11	48589864	0.000	0.02	0.23	PHAVU_011G206300g
	100050904	1	333838	0.000	0.02	0.21	PHAVU_001G003500g
	3378247	10	40113239	0.000	0.21	0.20	PHAVU_010G130800g
	8198264	3	29149401	0.000	0.01	0.19	PHAVU_003G116700g
	8211638	3	24713365	0.000	0.01	0.19	PHAVU_003G101200g
	8207991	3	31845700	0.000	0.03	0.18	PHAVU_003G129900g
	8206892	7	9596452	0.000	0.19	0.15	PHAVU_007G093800g
	100118766	4	40215749	0.00	0.25	0.14	
	3370846	6	12663395	0.00	0.18	0.14	
	8197486	11	50215927	0.00	0.05	0.14	PHAVU_011G216700g
	3379537	6	12895866	0.00	0.03	0.14	PHAVU_006G030700g
							PHAVU_006G030800g
PMR	3382116	9	8585442	0.00	0.47	0.13	
	3382360	9	10066070	0.000	0.20	0.30	PHAVU_009G053900g
	3377587	3	43928582	0.00	0.04	0.28	
	3382636	6	14690834	0.00	0.47	0.28	
DTF	3379537	6	12895866	0.000	0.03	0.48	PHAVU_006G030800g
	3370846	6	12663395	0.000	0.18	0.41	
	3379313	3	6715929	0.000	0.39	0.31	PHAVU_003G0533000g
	8207991	3	31845700	0.000	0.03	0.30	
	100103598	6	14910556	0.000	0.24	0.29	
	3380870	6	3275140	0.000	0.04	0.27	PHAVU_006G006900g
	3378348	2	42124682	0.000	0.41	0.26	
	3381588	9	24639921	0.000	0.39	0.24	PHAVU_009G168800g
	3378595	1	42919530	0.000	0.41	0.24	
	3383952	6	13738430	0.000	0.07	0.24	
	3377998	2	19829201	0.000	0.41	0.23	
DTM	3381766	2	623837	0.000	0.38	0.18	PHAVU_002G005400g
	3370833	7	41798767	0.000	0.38	0.17	PHAVU_001G137800g
	100118763	7	20337949	0.000	0.44	0.17	
	3369049	5	1228042	0.000	0.42	0.16	
	13121464	6	26586984	0.00	0.38	0.16	PHAVU_006G152900g
	100086666	7	39583133	0.00	0.24	0.15	PHAVU_011G093900g
	3384410	2	48039523	0.00	0.31	0.14	PHAVU_002G320900g
NPP	13122062	1	51811442	0.00	0.20	0.42	PHAVU_001G264500g
	3377146	3	50623920	0.000	0.02	0.41	
	8212460	2	30773757	0.000	0.40	0.41	PHAVU_002G166000g
	3379026	1	51926066	0.000	0.23	0.41	
	100049197	8	22139614	0.000	0.22	0.41	
	3381507	11	5055028	0.000	0.24	0.40	
	3383526	10	40587716	0.000	0.30	0.40	PHAVU_010G134100g
	13122571	11	25423374	0.00	0.39	0.39	
	100086011	1	15946014	0.00	0.27	0.39	
	3378689	8	7728249	0.00	0.27	0.39	
	3374909	11	3542136	0.00	0.41	0.39	
	3370570	3	33167524	0.00	0.37	0.38	
	3380620	11	3881871	0.00	0.41	0.38	
	8215091	10	2257189	0.00	0.14	0.38	
NSP	8175995	7	48190209	0.000	0.06	0.38	
	3372842	1	51394955	0.000	0.23	0.37	
	8669019	8	58703180	0.000	0.07	0.37	
	3378274	8	58679992	0.00	0.10	0.37	PHAVU_001G258000g
	100117354	7	18306669	0.00	0.35	0.36	
GYD	3377146	3	50623920	0.000	0.02	0.48	
	8215747	10	3143091	0.000	0.23	0.45	
	3369676	9	15099238	0.000	0.41	0.45	
	3381188	1	6465142	0.000	0.39	0.44	
	3383503	10	3012756	0.000	0.31	0.44	PHAVU_010G019700g
	8207731	6	12530595	0.000	0.15	0.44	
	3383465	10	3838534	0.000	0.03	0.44	PHAVU_009G099000g
	3377061	11	1164972	0.000	0.07	0.44	
	100053883	8	46259025	0.000	0.39	0.43	
	3381982	2	32037784	0.000	0.39	0.43	
	13122571	11	25423374	0.00	0.39	0.43	
	3383223	2	31567682	0.00	0.39	0.43	PHAVU_001G245500g
	100103590	8	47186185	0.00	0.34	0.43	
	8215249	1	1636953	0.00	0.23	0.43	
	8216463	4	44160933	0.00	0.48	0.43	PHAVU_004G159900g
	100116632	10	34395982	0.00	0.36	0.42	
	3365631	10	41170848	0.00	0.42	0.42	
	3384349	1	50470209	0.00	0.38	0.42	
	3383570	1	8000131	0.00	0.41	0.42	PHAVU_001G064100g
	3378416	2	31532508	0.00	0.17	0.42	

BDS = bean fly damage severity, PC = pupa count, PMR = plant mortality rate, DTF = days to 50% flowering, DTM = days to 90% physiological maturity, NPP = number of pods per plant, NSP = number of seed per pod and GYD = grain yield.

**Fig 5:**
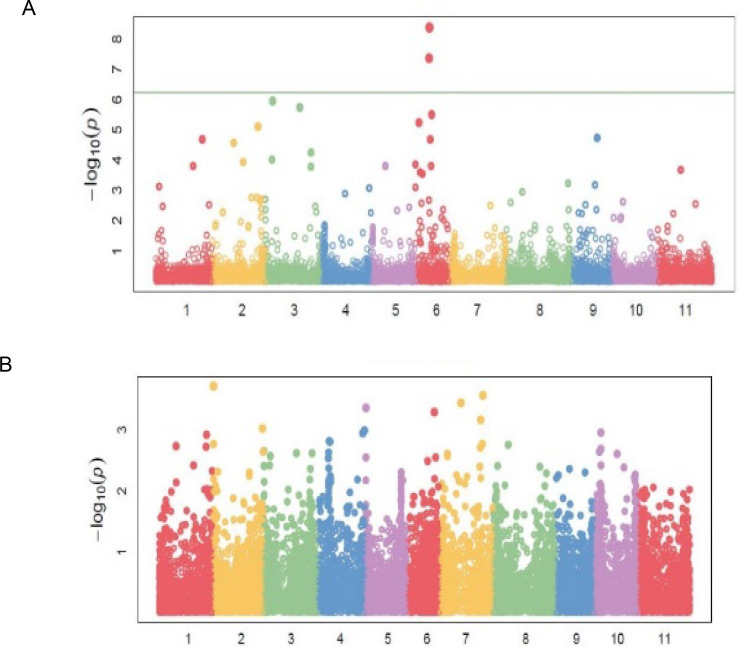
Manhattan plots showing candidate single nucleotide polymorphism and their probability values from genome wide association of 99 common bean genotypes assessed in two locations (Chitedze and Mbawa research stations) and two years (2018 and 2019) in Malawi. Note: A = Days to 50% flowering (DTF), B = Days to 90% physiological maturity (DTM).

**Fig 6:**
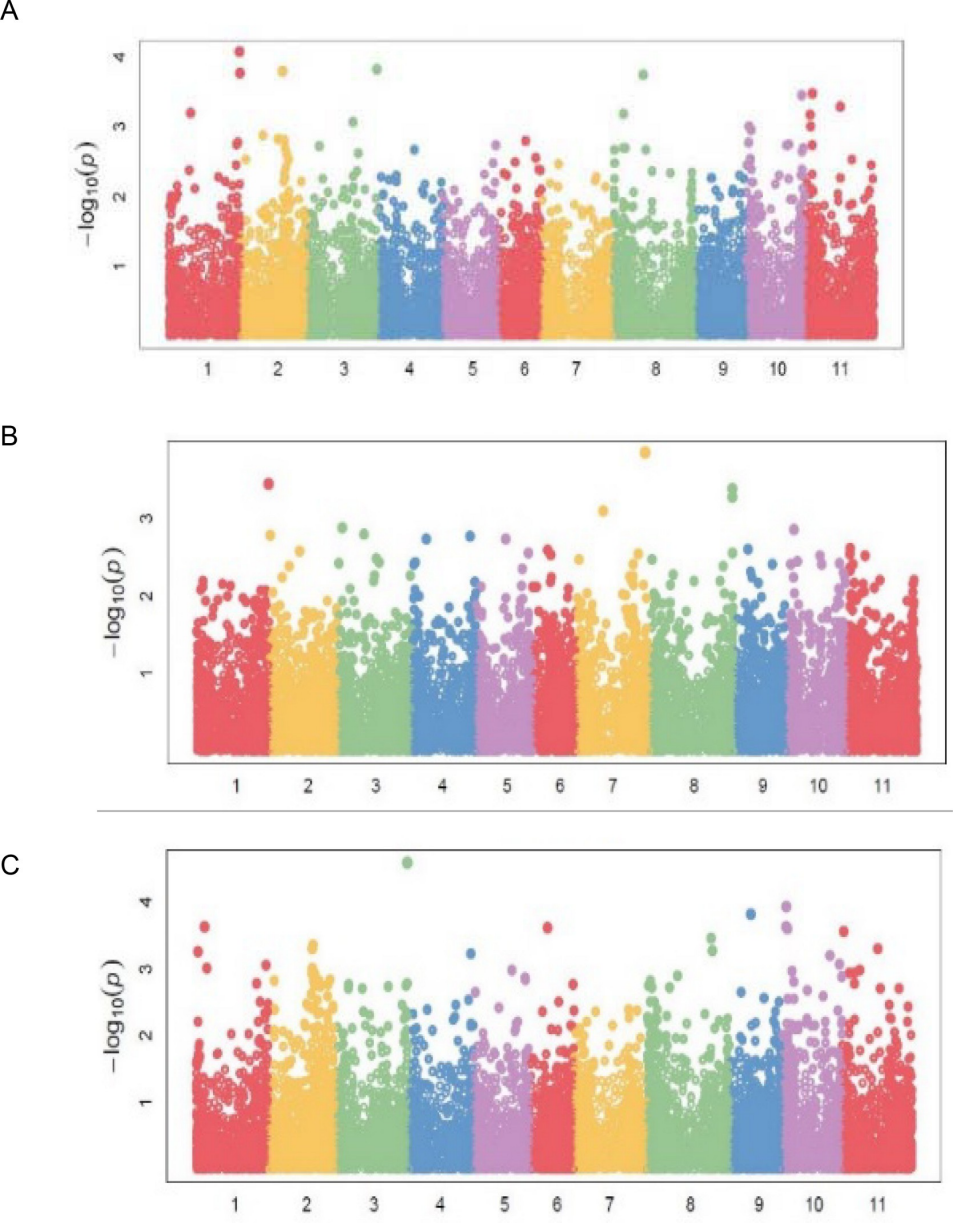
Manhattan plots showing candidate single nucleotide polymorphism and their probability values from genome wide association of 99 common bean genotypes assessed in two locations (Chitedze and Mbawa research stations) and two years (2018 and 2019) in Malawi. Note: A = Number of pod per plant (NPP), B = Number seed per pod (NSP), C = Grain yield (GYD).

Markers associated with DTF were distributed on chromosomes *Pv01*, *Pv02*, *Pv03*, *Pv06*, and *Pv09* (Table 4; Fig 5A). Chromosome *Pv06* had five markers, followed by *Pv03* and *Pv02* with two markers each. Markers *M337537* and *M3370846*, both on *Pv06*, exhibited the strongest associations with DTF with respective R^2^ values of 48 and 41 percent (Table 4). Significant markers for DTM were located on *Pv02*, *Pv05*, *Pv06*, and *Pv07*, while the 14 markers for NPP were distributed on six chromosomes (Table 4; Fig 5B). Grain yield had the highest number of markers [22] distributed in all chromosomes except on *Pv05* and *Pv07* (Table 4; Fig 6C). Chromosome *Pv10* had five markers, followed by *Pv04* and *Pv01*, which had four markers each. Marker *M3377146* on *Pv03* exhibited the highest association with GYD (R^2^ = 0.48). Two pleiotropic markers affecting BDS and GYD were identified on *Pv01* and *Pv10*. Markers *M100049197*, *M3379537*, and *M13122571* were pleiotropic for BDS and NPP, DTF and PC, and NPP and GYD, respectively.

### Linkage disequilibrium for significant markers

**Fig 7:**
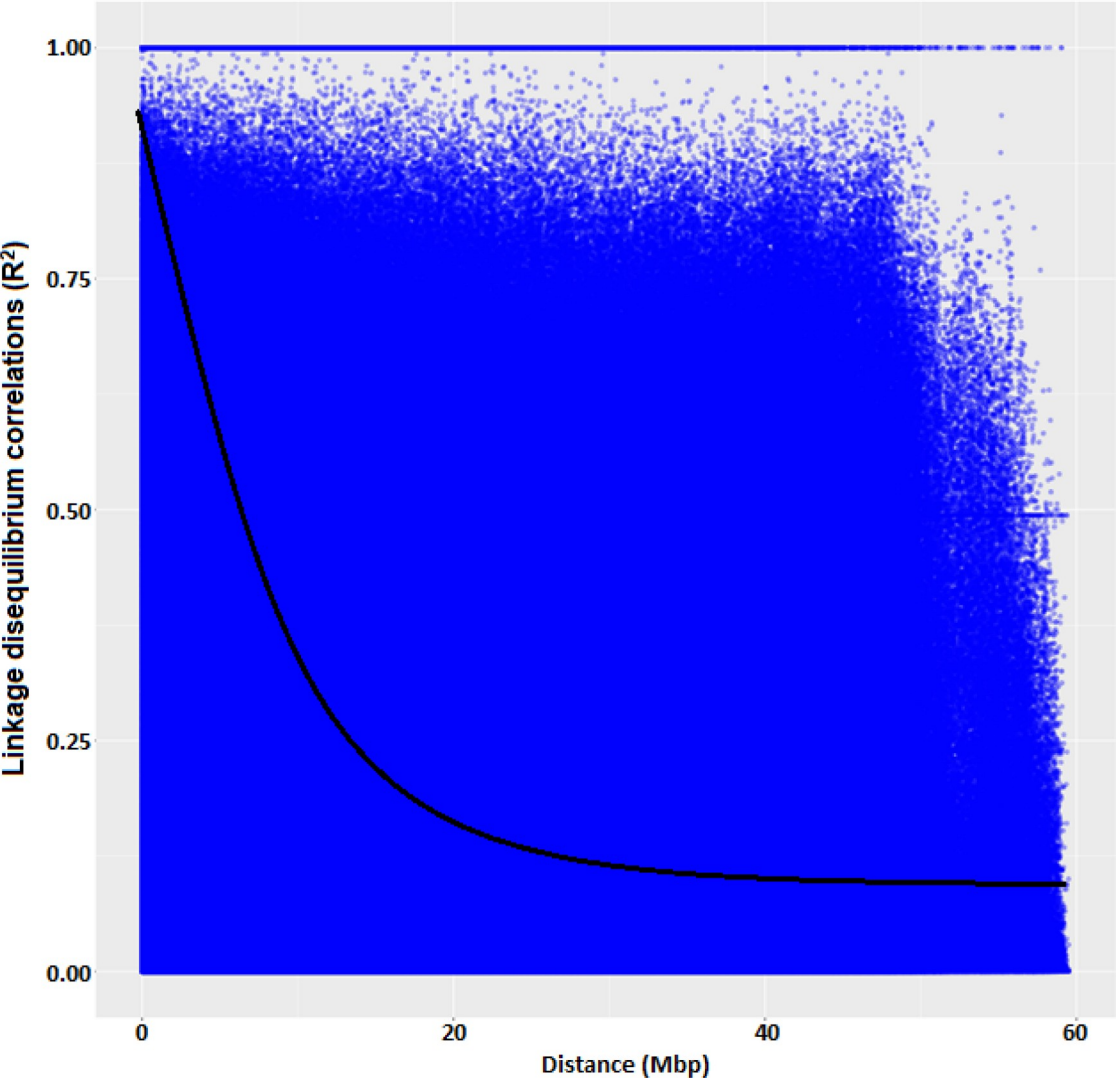
Linkage disequilibrium (R2) plot for all the 16,565 SNP markers across genome in common bean genotypes used in the study.

**Fig 8:**
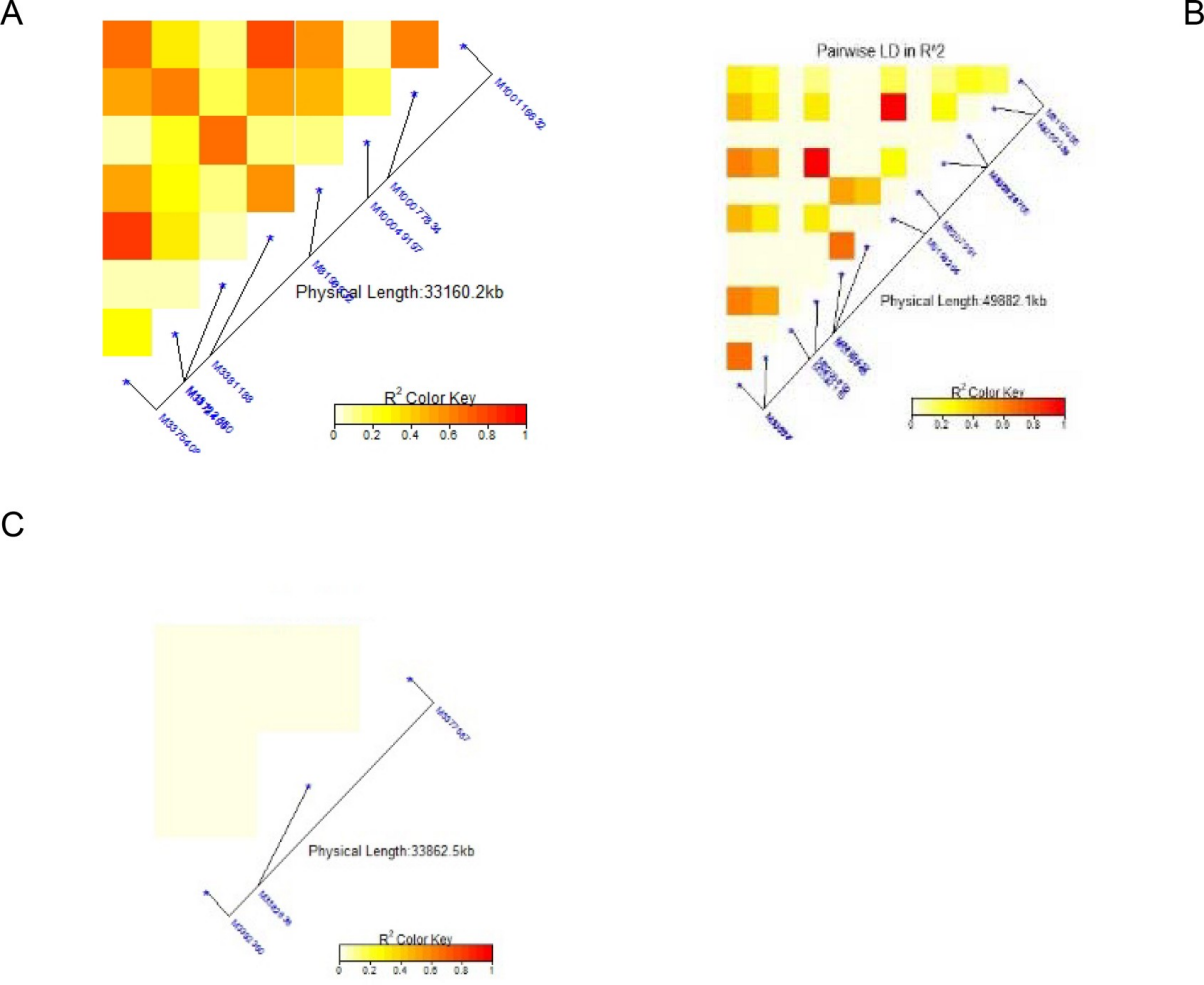
Summary of local LD among markers with significant marker trait associations of 99 common bean genotypes. Note: A = Bean fly damage severity (BDS), B = Pupa count (PC), C = Plant mortality rate (PMR). The R^2^ colour key indicates the degree of significant association.

**Fig 9:**
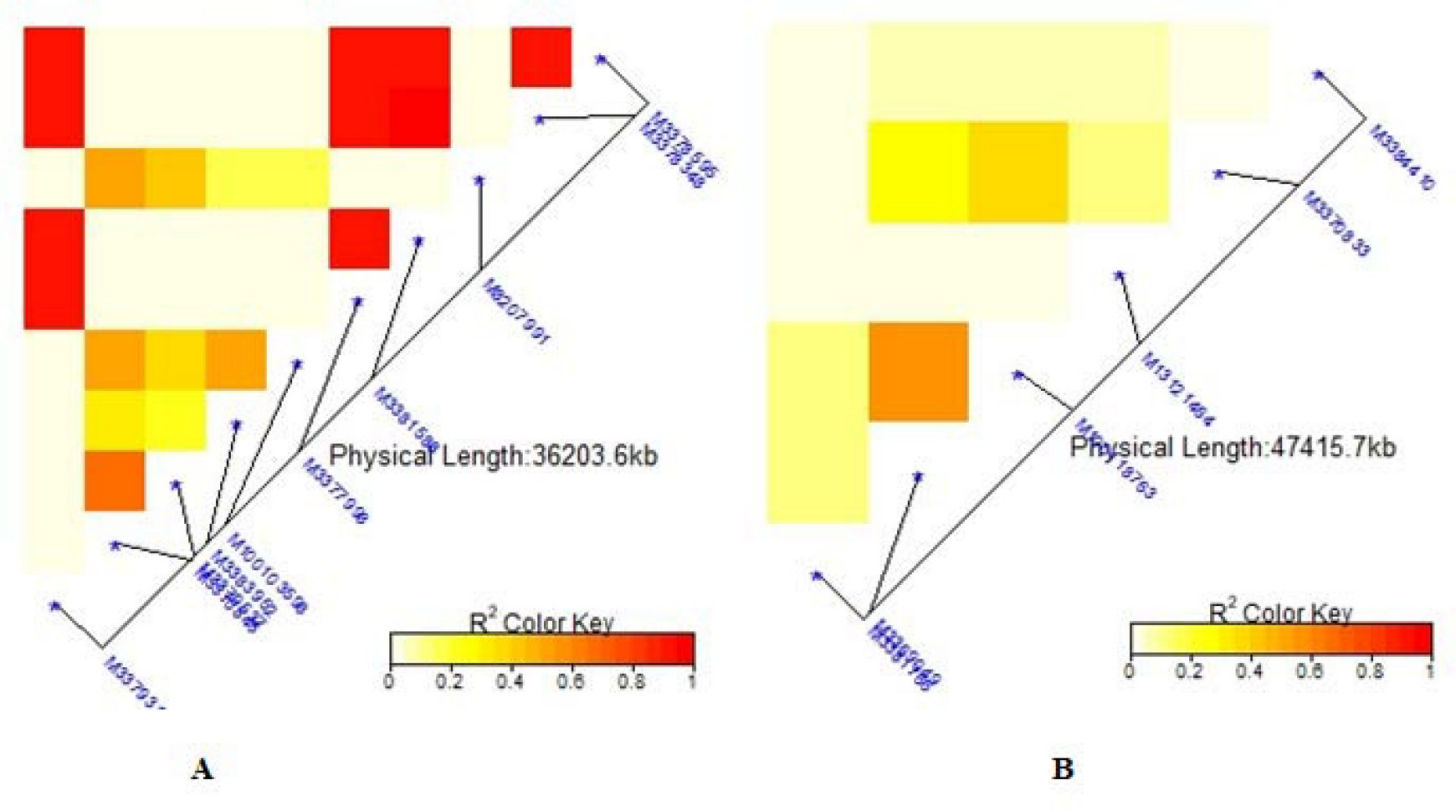
Summary of local LD among markers with significant marker trait associations of 99 common bean genotypes. Note: A = Days to 50% flowering (DTF), B = Days to 90% physiological maturity (DTM). The R^2^ colour key indicates the degree of significant association.

**Fig 10:**
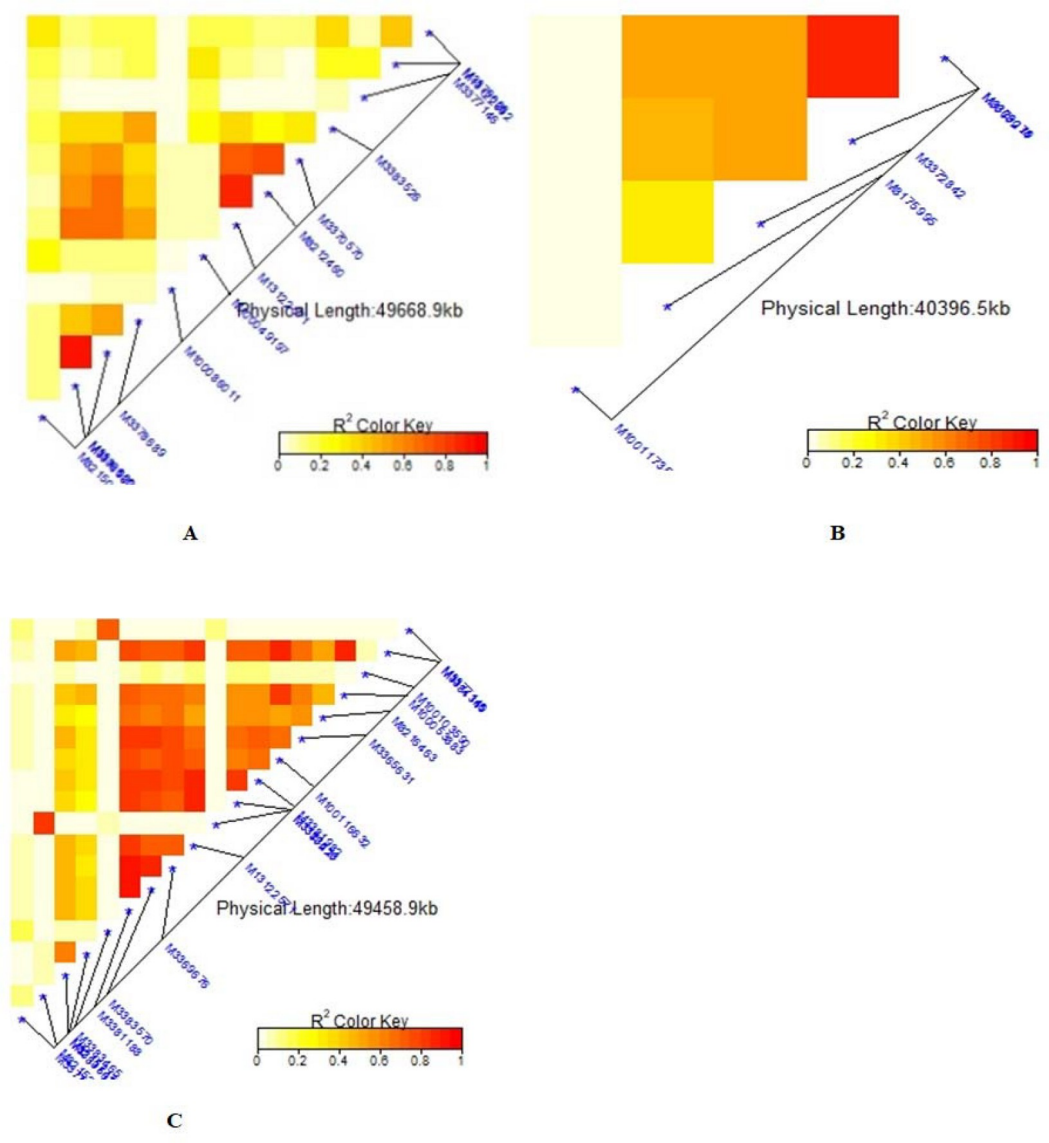
Summary of local LD among markers with significant marker trait associations of 99 common bean genotypes. Note: A = Number of pod per plant (NPP), B = Number seed per pod (NSP), C = Grain yield (GYD). The R^2^ colour key indicates the degree of significant association.

The average linkage disequilibrium (R^2^) across the whole genome was 0.20, which occurred at a distance of about 22Mb (Fig 7). The LD for individual traits ranged from very weak correlations (r<0.20, p<0.001) to very strong correlations (r>0.08, p<0.001) (Figs 8A–8C, 9A, 9B and 10C). Among the bean fly resistance traits, markers associated with BDS occurred within the shortest genetic distance of about 33Mb, although the R^2^ values ranged between 0.20 and 0.60 (Fig 8A). The linked markers were on chromosomes 6, 8, and 10. In comparison, PC had 12 markers that spanned a distance of 49.8Mb, and two of its associated markers had a strong correlation (R^2^>0.70) (Fig 7B). The markers were spread across chromosomes 3, 6, 10, and 11. Three markers associated with PMR spanned a genetic distance of 33.8Mb but exhibited weak association values (R^2^<0.20) (Fig 7C). These markers were on chromosomes 6 and 9. The genetic distance for markers associated with DTF (Fig 9A) was shorter (36.2Mb) than for DTM (47.4Mb) (Fig 9B). NPP had 10 markers that spanned on a genetic distance of 36.2Mb, and half of these exhibited stronger correlations (R^2^>0.70) (Fig 10A). Markers, *M10011735*, *M3175996*, and *M3372842*, were associated with NSP, and these spanned a genetic distance of 40.4Mb. Grain yield had 22 markers that spanned a distance of 49.5Mb, and 12 of these markers exhibited a strong correlation (R^2^>0.70) (Fig 10C).

### Putative candidate gene analysis

A blast search of potential bean fly resistance candidate genes identified 11 genes for PC and one for PMR (Table 3). Candidate genes for PC were identified on chromosomes *Pv01*, *Pv03*, *Pv05*, *Pv06*, *Pv07*, *Pv10*, and *Pv11*. Of the identified genes, three were on *Pv03*, and two were on Pv11. Two genes, *PHAVU_006G030700g* and *PHAVU_006G030800g* (annotated as protein-coding genes) were linked to marker *M3379537* on chromosome *Pv06* associated with PC. For PMR, gene *PHAVU_009G053900g* was linked to marker *M3382360* on chromosome *Pv09*. For DTF and DTM, four and five genes were blasted, although the annotated functions for these genes could not be established. Three candidate genes, including the adenosine triphosphate binding gene *PHAVU_002G166000g* were identified for NPP. Five candidate genes for GYD were identified, with *Pv10* having the two genes, including *PHAVU_009G099000g* (annotated as zinc ion binding). The other genes *PHAVU_001G064100g*, *PHAVU_001G245500g*, and *PHAVU_004G159900g* were identified on *Pv01*, *Pv02*, and *Pv04*, respectively.

## Discussion

The significant impact of genotype × location × year interaction effects on bean fly resistance parameters and agronomic traits suggests considerable genetic variation among the genotypes, and genotype performance exhibited environmental plasticity. Genotype × environment interaction results from variable and inconsistent genotype reaction to different seasonal and location conditions. This could be the basis for identifying genotypes with superior and specific adaptation to different environments. These results support earlier studies that there is potentially adequate genetic variation for bean fly resistance and agronomic traits to support breeding efforts [19,20]. The wide variation would help improve bean fly resistance and other traits in common bean through conventional and molecular breeding. The wide variation for DTF and DTM would permit the selection of genotypes in different maturity groups. In contrast, the genetic variation for NPP and NSP would be important for indirect selection for grain yield improvement. Indirect selection of yield-related traits is common practice for yield improvement in many crops [54].

Despite a lack of complete resistance to bean fly among the genotypes in the studied population, breeding lines exhibited a higher level of resistance than landraces and released varieties. Breeding for bean fly resistance is one of the important objectives in common bean breeding programs worldwide. Most breeding lines used in this study have likely undergone deliberate breeding for improved bean fly resistance at some stage in the breeding cycle. Likely, bean fly resistance genes from lines such as A429 and A55 have been introgressed into the breeding lines during their development [55]. Ambachew et al. [17] also found that breeding lines were more tolerant to bean fly infestation than released cultivars. Possibly bean fly resistance present in the released varieties and cultivars was based on a similar resistance mechanism leading to the broken down of resistance by the bean fly. The Mesoamerican lines exhibited higher resistance than the Andean genotypes suggesting that the Mesoamerican gene pool would be more useful in breeding for bean fly resistance. This study’s findings corroborate with previous reports showing that Mesoamerican genotypes tended to be more tolerant to the majority of biotic and abiotic stresses than the Andean genotypes [17].

The test genotypes were grouped into two sub-populations, suggesting that the clustering was based on the genotypes’ genetic ancestry. This confirmed previous assertions that common bean evolved from the Mesoamerican and Andean gene pools [56,57], which greatly influence genetic diversity in common bean. The population structure analysis even grouped the different botanical types (breeding lines, cultivars, and released varieties) into their broad gene pools showing that the evolution events were stronger than the subsequent recombinations that occurred in modern breeding. Similarly, germplasm from different centres of diversity, such as the ESA region, has been identified as distinct from the Mesoamerican and Andean gene pools [3]. However, the Mesoamerican and Andean gene pools are still profoundly detectable at the genomic level leading to clustering of genotypes along the broad Mesoamerican and Andean gene pool lines [35,58]. The PCA also grouped the genotypes into two groups congruent to the population structure analysis, highlighting the broad gene pools’ importance. This limit selection of divergent parental lines and breeding gains. Parental breeding lines can only be selected within the two gene pools, limiting allelic diversity since allele frequency is constrained within a population of related individuals. The genetic structure obtained in the study was consistent with the hierarchical clustering of common bean genotypes into two major groups based on Mesoamerican and Andean origins [35,59,60]. The majority of the evaluated lines were of Andean origin, which supported previous studies reporting that most African common bean germplasm was of Andean origins [3,61]. The Andean gene pool is the source of most African germplasm and could contribute to restricted breeding gains with intra-gene pool crosses. Repoterdly, the Andean gene pool had limited genetic bases before domestication [62]. The evidence of genetic constraints in this gene pool could delay genetic gains in breeding for polygenic traits such as improved bean fly resistance. Crossing parental lines from different gene pools could potentially eliminate the genetic bottlenecks. For instance, the BDS and PMR were relatively higher, which corresponded to lower bean fly resistance in the Andean genotypes compared to Mesoamerican gene pools. This points to the high likelihood of crosses derived from Andean parental lines having lower resistance due to their genetic background. The low mean MAF of 0.23 found in this study suggested a limited number of rare variants among the accessions, indicating that most genotypes shared common alleles [63]. Rare variants have been implicated in playing a bigger role in complex traits such as disease resistance [64]. The low MAF showed that the genetic base for developing bean fly resistance might need to be widened to increase the rare variants that potentially code for resistance. Targeted crosses and mutation induction have the potential to increasing the frequency of rare variants and widen the genetic base of the current population. The narrow population structure, low heterozygosity and MAF values in this germplasm suggests the need for controlled crosses involving genetically unique populations with economic traits to broaden the genetic variation. There were few admixtures identified in the germplasm, which was expected since varietal mixtures in the common bean are common in Malawi and other ESA countries. This is due to mixed cropping practices,limited knowledge pedigree of bean types, and a lack of true-to-type varieties [60]. The average PIC was 0.25, indicating that this study’s SNP markers were low to moderately informative. The low PIC value indicated that the germplasm’s genetic base was relatively narrow or moderate, which limits the possible permutations during crossing. At the genotypic level, the PIC values show that genetic variation was moderate. The phenotypic differences observed in the population would be more defined by genotype × environment interaction than genotypic differences. The low average PIC could be due to SNP makers’ bi-allelic nature, which restrict PIC values to ≤ 0.5 [65,66]. In general, markers such as SNPs usually have less PIC values than simple sequence repeats [67]. However, SNP markers provide a means for identifying allelic diversity at numerous loci [68,69], making them more useful in genetic diversity and genomic analyses.

The markers associated with bean fly resistance were evenly distributed across the 11 chromosomes, indicating that bean fly resistance is a polygenic trait controlled by major and minor genes distributed across the whole genome [23,24]. Improvement of bean fly resistance will be complicated by the whole genome distribution of the involved markers and may involve several cycles of breeding to fix the genes. Unlike Mendelian traits whose inheritance can easily be predicted, the inheritance of polygenic traits such as bean fly resistance is difficult to predict. It would require efficient phenotyping in multiple environments to identify stable genotypes that exhibit resistance. The average R^2^ of 0.20 showed that there was a moderate to weak linkage among some of the markers that were inherited together. Linkage disequilibria occur when markers are inherited, leading to the distortion of haplotypes’ expected frequencies [70]. The occurrence of disequilibria at 22Mb shows that the LD events occurred at relatively short intervals, which would be unexpected for an inherently autogamous species such as common bean in which recombinant events take place less frequently compared to outcrossing species. However, the presence of elite breeding lines and released cultivars could have reduced the LD distance due to accelerated recombination during their development using divergent parental lines. Also, shorter LD decay is possible because common bean usually exhibits large blocks of markers in LD despite being an autogamous species [71]. Rapid LD decay is important for the recombination and development of new recombinants to improve adaptation and potential identification of resistant genotypes. The LD found in this study was comparably higher than 10 Mb reported by [72] in common bean germplasm that included commercial cultivars, landraces, and recombinant inbred lines (RILs) derived from the cross ‘CAL 143’ × ‘IAC UNA’. This relatively fast LD decay results from more recombination events in inherently self-pollinating species especially considering that the germplasm included breeding lines, released varieties and Mesoamerican genotypes. Several other reports have alluded that populations derived from Meso-American gene pool had faster LD decay compared to the Andean gene pool [73,74]. Markers for BDS and PC were identified on *Pv01*, similar to reports by Ojwang et al. [36]. This suggests that chromosome *Pv01* harbours genes controlling bean fly resistance. The present study has also identified additional markers associated with bean fly resistance on other chromosomes that were not previously reported, which could be novel loci that require validation. Chromosomes *Pv06*, *Pv09* and *Pv10* contained several markers for BDS, PC and PMR in linkage disequlibrium showing that these chromosomes could be important for mining alleles for improving bean disease resistance. Chromosomes Pv09 and Pv10 have been implicated as harbouring genes involved in main characteristics such as fowering time, plant size, and seed size used during selection for domestication [71]. Markers associated with bean fly resistance were also associated with other traits such as DTF and GYD. These pleiotropic markers could be important for multiple trait selection in developing bean fly-resistant cultivars that are high-yielding and early maturing. Pleiotropism has been reported in other association studies on common bean [75,76]. Blasting for candidate genes for bean fly resistance traits revealed several candidate genes for PC and only one candidate gene for PMR. The majority of these were protein-coding genes. Protein coding genes have been reported to be important in disease and pest resistance in common bean [34,77].

The study found significant markers for DTF on chromosomes *Pv01*, *Pv03*, and *Pv06*, which corroborated previous reports for the same trait [28,29,78]. This suggests that the markers are stable across different populations and in different environments, essential for marker-assisted breeding for early maturity. Additional markers were identified associated with DTF on chromosomes *Pv02* and *Pv09* that were not previously reported. The new markers need to be validated in subsequent studies.A blast search identified four candidate genes for DTF on *Pv03*, *Pv06*, and *Pv09*, which was in agreement with previous studies [28,79]. Among the identified genes were protein-coding genes such as *PHAVU_009G168800g*, *PHAVU_006G006900g*, and *PHAVU_006G030800g*. The *PHAVU_009G168800g gene belongs to the* Mu1/VP4 superfamily of genes involved in host cell surface binding [80]. The gene *PHAVU_006G006900g* is involved in selective and non-covalent interaction with zinc ions and nucleic acid binding [81]. Protein coding genes have been important in regulating flowering time in common bean [28]. Markers for DTM were reported on five chromosomes, including *Pv07*. Moghaddam et al. [82] reported markers for DTM on *Pv07*, suggesting that genomic regions control maturity on chromosome *Pv07*.

Markers associated with NPP and NSP were identified on chromosomes *Pv07* and *Pv11*, suggesting the presence of genomic regions associated with seed-related traits on these chromosomes. Blair et al. [83] reported QTL for NPP and NSP on *Pv11* and *Pv07*, respectively. Grain yield markers were identified on all chromosomes except *Pv05* and *Pv07*, suggesting that grain yield is polygenic. Numerous genes condition yield with minor genetic effects [84]. Previously, markers for GYD were also identified on chromosomes *Pv03*, *Pv08*, *Pv09*, and *Pv10*, which indicate the stability and potential usefulness of these markers for marker-assisted breeding [29]. Also, markers such as *M100116632*, *M3381188*, *M3379537*, and *M8207991* exhibited pleiotropic effects on different traits. Pleiotropic markers could be useful to select for multiple traits simultaneously. Markers with pleiotropic effects have been previously identified and used in common bean breeding. For instance [29], found a pleiotropic marker for NPP and GYD, while [85] found that DTF and DTM shared a similar marker. Five candidate genes were identified for GYD from the significant markers, and the molecular function of *Phavul_009G099000g* was annotated as zinc ion binding. Zinc is an important constituent of hormones and is involved in internode elongation [86]. These functions are important for plant growth and development, and internode development is particularly critical in common bean. Growth and development directly impact grain yield production in common bean [87]. The lack of adequate Zn can lead to significant yield loss and plant death [88], and thus the marker can be used for selecting genotypes with enhanced ability to bind zinc.

## Conclusion

The assessed common bean population exhibited significant variation for agronomic and bean fly resistance traits, which enabled GWAS to be conducted successfully. The 83 markers detected across the genome indicated both agronomic and bean fly resistance traits were conditioned by multiple genes, some with pleiotropic effects. Two markers, *M3381188* and *M100116632* with pleiotropic effects for BDS and GYD, were respectively identified on chromosomes *Pv01* and *Pv10*. These are effective markers for simultaneous improvement of bean fly resistance and grain yield in common bean. A weak R^2^ value of 0.2 that occurred at a distance of 22Mb was detected, indicating that most of the markers were not tightly linked and could be selected independently. The present study identified novel markers (e.g. *M3381188*, *M338320*, *M33753*, *M3370846*, and *M3377146*) for marker-assisted breeding in common bean.

## Supporting information

S1 Fig Quantile-quantile plots indicating the normality of 99 common bean genotypes assessed in two locations (Chitedze and Mbawa research stations) and two years (2018 and 2019) in Malawi. Note: A = Bean fly damage severity (BDS), B = Pupa count (PC), C = Plant mortality rate (PMR).
(TIF)

S2 Fig Quantile-quantile plots indicating the normality of 99 common bean genotypes assessed in two locations (Chitedze and Mbawa research stations) and two years (2018 and 2019) in Malawi. Note: A = Days to 50% flowering (DTF), B = Days to 90% physiological maturity (DTM).
(TIF)

S3 Fig Quantile-quantile plots indicating the normality of 99 common bean genotypes assessed in two locations (Chitedze and Mbawa research stations) and two years (2018 and 2019) in Malawi. Note: A = Number of pod per plant (NPP), B = Number seed per pod (NSP), C = Grain yield (GYD).
(TIF)

S1 Data Genotypic data. (XLSX)

S2 Data Phenotypic data. (XLSX)
