# Supplementary figures and images for: Genome-wide association analysis of bean fly resistance and agro-morphological traits in common bean
Source: PLoS One. 2021 Apr 29;16(4):e0250729. doi: 10.1371/journal.pone.0250729 (PMC8084209; doi:10.1371/journal.pone.0250729)

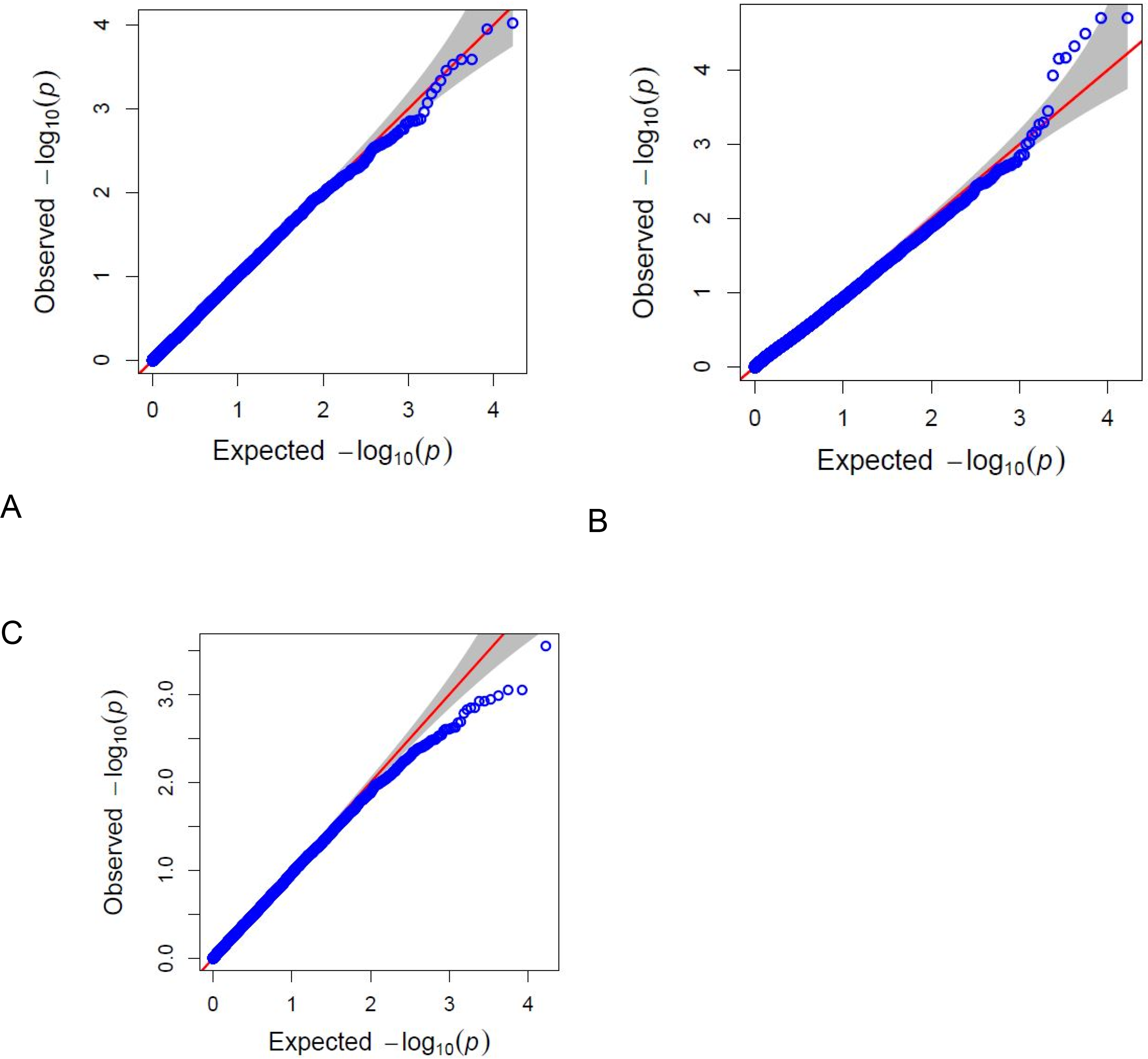

Supplement: S1 Fig — Note: A = Bean fly damage severity (BDS), B = Pupa count (PC), C = Plant mortality rate (PMR). (TIF) [file pone.0250729.s001.tif]

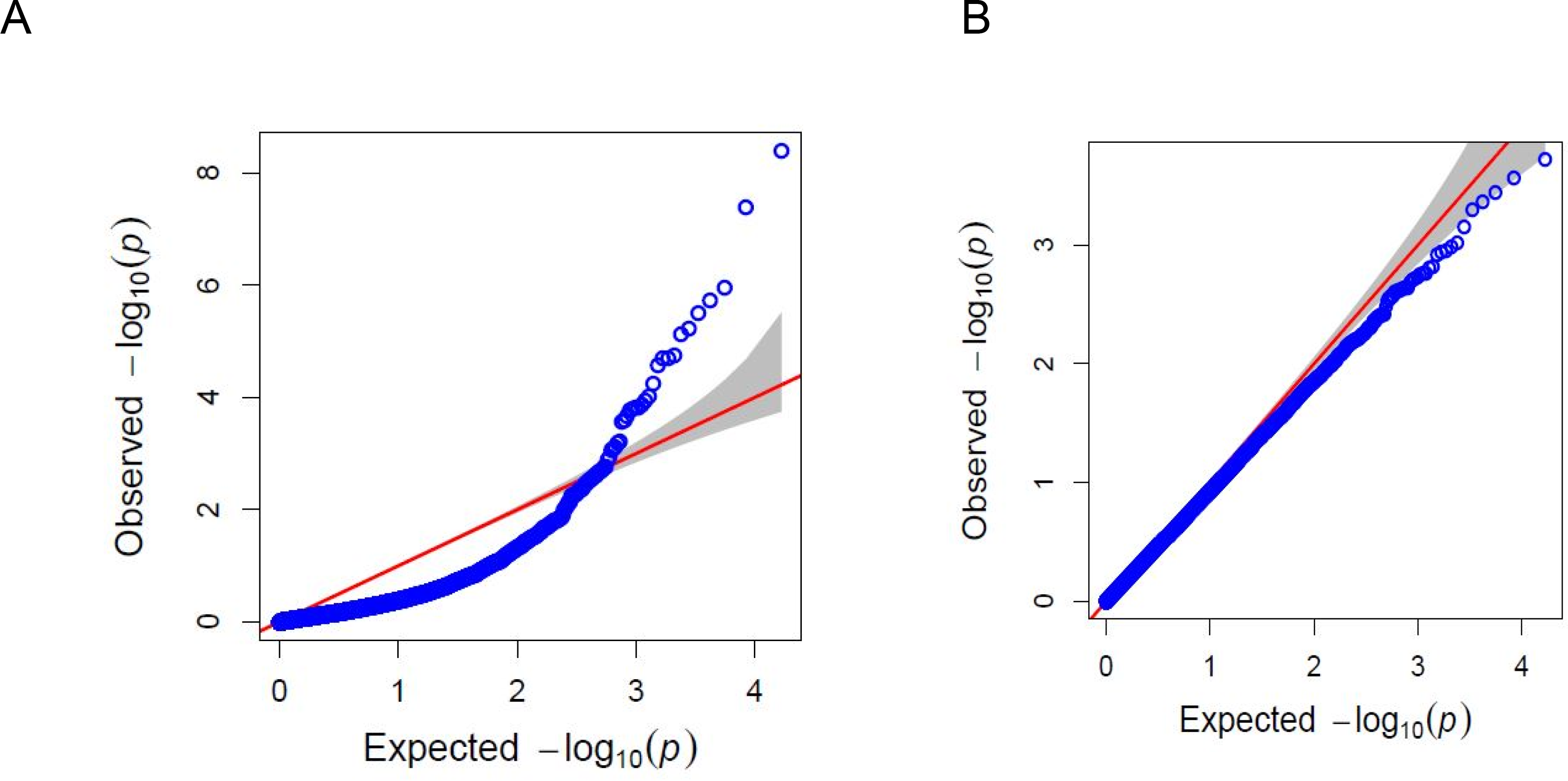

Supplement: S2 Fig — Note: A = Days to 50% flowering (DTF), B = Days to 90% physiological maturity (DTM). (TIF) [file pone.0250729.s002.tif]

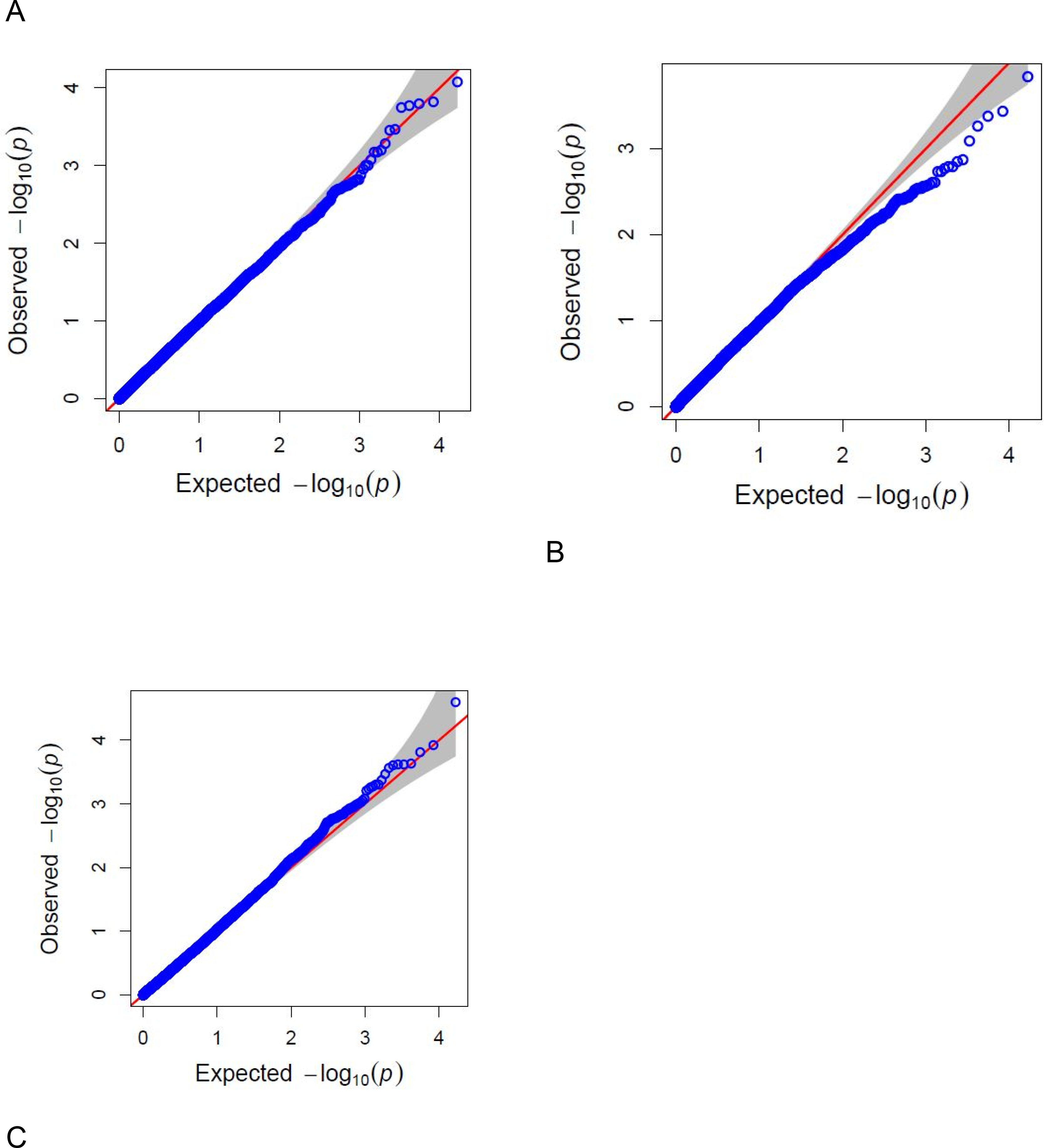

Supplement: S3 Fig — Note: A = Number of pod per plant (NPP), B = Number seed per pod (NSP), C = Grain yield (GYD). (TIF) [file pone.0250729.s003.tif]
